# Scientific and technical data sharing: a trading perspective

**DOI:** 10.1007/s10822-014-9785-4

**Published:** 2014-08-12

**Authors:** Jeremy G. Frey, Colin L. Bird

**Affiliations:** Chemistry, University of Southampton, Southampton, SO17 1BJ UK

**Keywords:** Perspective, Viewpoint, Data, Sharing, Trading, Exchange, Broker

## Abstract

It is arguably a precept that the open sharing of data maximises the scientific utility of the research that generated that data. Indeed, progress depends on individual scientists being able to build on the results produced by others. The means to facilitate sharing undoubtedly exist, but various studies have identified reluctance among researchers to share information with their peers, at least until the professional priorities of the original researchers have been accommodated. With a view to encouraging less inhibited collaboration, we appraise the processes of data exchange from the perspective of a trading environment and consider how data exchanges might promote (or perhaps hinder) collaboration in data-rich scientific research disciplines and how such an exchange might be set up. We suggest an exchange with trusted brokers (akin to the commodity markets) as a way to overcome the challenges of the current environment. We conclude by encouraging the scientific and technical community to debate the merits of a trading perspective on data sharing and exchange.

## Introduction

It is arguably a precept that the open sharing of data maximises the scientific utility of the research that generated that data [1]. Indeed, progress depends on individual scientists being able to build on the results produced by others [2]. The means to facilitate sharing undoubtedly exist, but various studies have identified reluctance among researchers to share information with their peers, at least until the professional priorities of the original researchers have been accommodated. However, there is little evidence of an integrated approach to the establishment of models that encourage open sharing and exchange, notwithstanding the appeals in the Royal Society report [1]. In this viewpoint article we consider the context and environment in which science tends to be conducted and put forward a model for integrating the channels for sharing and exchange, focusing particularly on the chemistry domain, while recognising fully the potential for wider application.

### Terminology

 In the context of scientific collaboration, the terms *sharing* and *exchange* are used almost interchangeably, thereby disregarding the wider understanding that *exchange* involves receiving something in return, whereas *sharing* might be more altruistic. Such distinctions often become blurred, in that *sharing* can be mutual and exchanged items are not necessarily equivalent in value. In the scientific research context specifically, neither *sharing* nor *exchange* involves an explicit assumption of receiving assets in return, apart from a reliance that proper attribution, such as citation, will be given.

We use the terms *sharing* and *exchange* interchangeably and in conjunction in this article, adopting a broad interpretation that each activity involves an individual or group making data and other collateral available for other researchers to use.

In this article, we also refer to open sharing, open access, open data, and openness in general. The Royal Society report [1] does not venture a definition of the term *open*, but does very effectively convey the widely accepted understanding of the meaning of the term, which we in turn adopt.

Trade is a synonym of *exchange*, with the subtle implication that the *trading* process involves some form of regulatory procedure. *Trading* commonly involves an agent of some form; we assess in a subsequent section the potential role of an agent in *trading* scientific and technical data.

### Context and environment

We are presenting a viewpoint with regard to the sharing of data that is generated as part of an experiment or other activity, in contrast with data created with the express intention of sharing it for a specific purpose. Our criterion for making such a distinction when defining our context is one of *purpose*.

For example, the Cambridge structural database (CSD) is “the world’s repository of experimentally determined organic and metal–organic crystal structures” [3]. Crystallographers determine these structures with the *purpose* of depositing the data to the CSD.

This article relates to facilitating the exchange of the substantial amounts of data generated as an adjunct to research with a *purpose* other than data creation. One example of such data is the spectral information obtained when confirming the structure of a synthetic product. Typically, the interpretation of the spectra would be included with the published report of the synthesis, but the raw spectral data would remain private. The context of this article is the sharing of complementary data, which is commonly retained in a variety of institutional and private stores.

That range of stores is a primary feature of the environment in which complementary data is generated and preserved. Data might be stored in a national or subject area repository, an institutional (university) repository, a web-based store such as Zenodo [4] and Figshare [5], a laboratory repository, a personal computer, and even on a flash drive or other portable media. Data might also be preserved with a publication, as supplementary material. The preservation environment is therefore complex and indicative of diverse means whereby shared data can be accessed.

Undoubtedly a considerable amount of data exchange and sharing occurs on a peer-to-peer basis, relying on pre-existing relationships. This article does not explore such informal exchanges, which would not in any case be susceptible to more formal processes.

The environment also comprises mechanisms that support access to data, for example DataCite DOIs [6] and Amazon Glacier Archive IDs [7]. The former could be regarded as an emerging standard.

Tracking data accesses and reuse thereafter is a more haphazard process, although tools are increasingly becoming available. In all cases, however, the onus is on the researcher to check for access and reuse. Google Scholar [8] has a provision for tracking citations to publications and researchers can deploy ImpactStory [9], for example, to measure and share their research impacts. Identifiers such as ORCID and ResearcherID enable the unique identification of researchers. The most significant challenge in tracking access and reuse is to establish with some certainly whether data has been reused or repurposed or obtained merely as a matter of interest or even curiosity.

In some instances, researchers will impose restrictions on the use of data that they are otherwise willing to share, the best-known example being the embargo placed on items until a given date, usually a publication date. Other forms of regulation are copyright licenses such as the Creative Commons license [10] and specific requirements applied by data providers, such as requests for feedback.

In presenting our viewpoint, we appraise the environment for exchanging and sharing data generated as a consequence of another activity. This environment comprises a disparate set of facilities that lack a means for linking them together, which we regard as a missing “hub” in an area of growing importance and activity. It is a pivotal point that the *hub* we envisage is not another data store: it is the embodiment of a mechanism for sharing data through a trusted broker that we believe has a well-founded analogy with a trading model. The broker would manage packages (manifests) that describe the data to be exchanged but would not manage or handle the data itself. The “currencies” involved in the trade are reward and recognition for the researcher.

We believe that the existence of a trusted broker operating a regulated data exchange mechanism would offer a valuable opportunity to the Higher Education Community. We further consider that a consortium of national and subject area repositories could advantageously operate the broker. We therefore offer our viewpoint to the community for discussion.

### The chemistry domain

We focus on the chemistry domain, while recognizing the indications that barriers to open access exist elsewhere in applied informatics. With regard to studies of information-seeking behaviour, Davis described chemists as an “ideal group to study”, owing to their heavy use of journal literature [11]. In the 10 years since that article was published, chemistry has become ever more dependent on data and accordingly on the preservation, curation, discovery, access, and provenance of that data. The authors of this article have recently reviewed information and data sharing in the chemical sciences from an e-Research perspective [12].

A survey of data sharing and the use of collaborative technologies by chemists revealed attitudes that appear to be inconsistent with a reliance on the published work of other researchers, such as a tendency to store data as hard copy and a reluctance to allow open access to research results [13]. Another article noted that chemists were not taking full advantage of Web-based resources, yet needed “unfettered bench-top access via the Web” [14]. A recent study of information sharing and exchange in the life sciences detected similar characteristics within that domain [15]. Bird, Willoughby, and Frey discuss attitudes to sharing and the attendant implications for record keeping in chemistry and other sciences in their review of laboratory notebooks in the digital era [16].

Discussing open access in the context of the scholarly publishing of data, Borgman noted that current practice tends to discourage data sharing and exchange [17]. She identified four categories of reasons for the reluctance of researchers to contribute data to repositories, which we summarise as follows:

- Reward systems favour publication rather than data curation;
- Significant effort is required to organise, manage, and curate data; we refer to this as the burden of curation;
- Research tends to be competitive, leading to a reluctance to share data until papers have been published and/or data is no longer commercially sensitive;
- Researchers value ownership of their data: it is their intellectual property.

We recognise that this potential minefield of conflicting interests and requirements requires a different perspective if the technical innovations of the Web and digital commerce are to help to increase the efficiency of scientific research by encouraging higher quality dissemination of data.

With a view to encouraging the growth of scientific and technical collaboration, we put forward a *trading environment* as a fresh perspective on scientific data sharing and exchange. We note that historically markets and exchanges evolved to address and manage the inhibitions and conflicts outlined in the preceding paragraphs, perhaps not always completely effectively but ideally at least transparently and with a clear audit trail.

Before we examine models for trading and exchange, we offer in the following section two illustrations of what can occur if data is not shared openly and in contrast what should happen.

### Motivations for open data sharing

In our introduction to the context and environment for this viewpoint article, we gave as an example of data generated as an adjunct to other research the spectral information obtained when confirming the structure of a synthetic product. We noted that, typically, the interpretation of the spectra would be included with the published report of the synthesis, whereas the raw spectral data would remain private.

The phrase “remain private” can conceal a range of potential inhibitors to the subsequent reuse and/or reinterpretation of that raw data. At best the data would have been preserved in an institutional (university) repository; at worst the data would have been retained on a personal computer or on portable media. The less controlled the storage medium, the greater the risk that the raw spectral data might subsequently be misplaced, making any reuse or reinterpretation impossible.

If the research group implements a robust data management policy, the raw spectral data would be held in an open access repository that has a warranted preservation period. The publication of the synthesis would be accompanied by a data citation, using for example a DataCite DOI.

## Models for trading and exchange

A trading infrastructure offers a novel social and technological solution based on economic models of exchange, which have evolved to ensure transparency, access, appropriate acknowledgement, and adherence to licence conditions. Exchanges (such as commodity and stock exchanges) can be found in all developed economies and once established, provide the reassurance necessary to encourage trading and to facilitate analysis, regulation, and accountability as required.

Among economic models of exchange, the closest correspondences to the sharing of scientific information are: (a) the gift economy, in which custom governs exchanges, rather than explicit remuneration contracts; and (b) knowhow trading, which is based on informal exchanges of technical knowledge. In these models the value of the commodity is not an explicit monetary value, but usually a rather more tenuous concept.

### Knowhow trading

Carter [18] describes knowhow trading as “the informal exchange of practical technical knowledge between pairs of engineers and other technicians in different firms”. Her approach is theoretical, whereas Meyer [19] relies on examples of “collective invention”. This term was originally defined by Allen [20] as “the free exchange of information about new techniques and plant designs among firms in an industry”. Meyer redefines the term as “a process in which improvements or experimental findings about a production process or tool are regularly shared”. Carter and Meyer set the scene, even though their context is commercial and related more to manufacturing rather than scientific data.

Communities can share technical information in ways ranging from patent licensing through collaborative projects to ad hoc groups with a common interest, such as the open source community. Knowhow trading lies in the middle of the sharing spectrum: it is based on established relationships. Meyer shows a presumption in favour of collective invention when outcomes are uncertain, noting that the “process evaporates” when the uncertainty diminishes.

Meyer introduces his paper [19] with the following distinction:

“Technological advances are often kept secret or patented, making them the intellectual property of their inventors. Scientific advances are more often published openly. One reason for the difference is that scientific investigation is driven so much by curiosity, whereas technological investigation is clearly functional, driven by the goal of producing something and usually to earn a profit.”

Von Hippel [21] characterized the behaviour he observed in steel minimill processing as: “an informal trading network that develops between engineers having common professional interests”. He distinguished knowhow trading from Allen’s view of collection invention in that the valuable information exchanged between traders remains secret from non-traders. On the other hand, Carter emphasises the practical nature of the technical knowledge and notes that it is “generally cheaper to acquire knowhow through exchanges”, even though competitive advantage can thereby be lost.

When involved in a trading network, scientific researchers and commercial technology developers would have in common not only a community with mutual interests but also a view of information and data as assets than could—optionally—be shared and/or exchanged, rather than as goods that have a market value. Moreover, while acknowledging that some advantage might be surrendered as a consequence of cooperation, a trading network should bring a recognition that cooperation is almost always cheaper (in saving of effort as well as in financial terms) than “going it alone”.

Knowhow trading is a form of barter, in which the partners exchange intellectual property assets, with an expectation that the assets are of approximately equivalent significance. In the scientific research context specifically, neither *sharing* nor *exchange* involves an explicit assumption of receiving assets in return, apart from a reliance that proper attribution, such as citation, will be given if recipients use the information or data to further their own work. In that sense, arrangements for scientific sharing would resemble a gift economy rather than knowhow trading. In effect, the existence of established relationships is a requirement of knowhow trading. Although data sharing is more likely to occur within an existing relationship, data can also shared on an ad hoc basis. As noted in the Introduction, the future progress of science depends on promoting a culture of sharing.

Arising from the broader basis just outlined, the selection of partners that is implicit in knowhow trading is not a necessary feature of sharing. With knowhow trading as described by Carter and von Hippel, the rewards are technological advance and saving the costs of “re-inventing the wheel”. When scientific data is shared, the rewards are imprecise and less certain: the advancement of knowledge and understanding; and the enhancement of professional reputations through citation.

The nature of the collaborating network is the difference that perhaps carries the most potential to be significant. Carter describes the emergence of knowhow trading networks as follows:

“Would-be traders develop networks of colleagues and get to know potential partners through professional organizations and informal referrals. Cumulative experience provides a basis for judging individual partners’ contributions.”

Although Carter rightly points out knowhow trading could extend into “multi lateral networks of three or more”, the nature of such networks would remain constrained by their constituent relationships. Meyer devotes an entire section to discussing the “social network perspective” of collective invention. Social networking support is vital for an information-sharing community, which can in practice be very fluid in nature.

### Trading scientific and technical data

Before we discuss whether scientists could—and should—trade in data, we consider briefly the extent to which data is, or can be, a part of “knowhow”. For example, if a process, procedure, or characterization relies explicitly on some data, that data forms part of the “knowhow”, and therefore becomes a component of the trade. Alternatively, if the data is no more than the result of applying a “knowhow” method, then consequential data is neither part of the knowhow nor a necessary component of any trade, although the data might be supplied gratuitously.

Information and any data associated with it are already traded routinely, one example being patent licensing, so the answer to the ‘could’ question has to be “yes”. Information differs from tangible goods in several ways, notably that information is held jointly rather than transferred. (Carter notes that special arrangements would be required to deny access to the original possessor of information).

The total holding of traded tangibles remains constant, whereas the sum of information holdings is a multiple of the number of possessors. Moreover, information has effectively no distribution costs, whereas the distance between suppliers and buyers of tangibles can influence trade, for example by encouraging entrepreneurs to set up markets in neutral locations;

Trade in tangibles usually occurs to offset imbalances: we trade our excess stock for other items that for us are in short supply and will be useful to us. When we trade information we do so only on the basis of its usefulness and not its quantity. The value of tangibles can derive from both intrinsic characteristics and usefulness; information has value only in terms of its usefulness. The value of information can vary between possessors and is fragile: one inappropriate transfer can destroy the usefulness of information and therefore its value.

We conclude that scientific data could be traded, provided we take due account of the preceding considerations. However, we argue that scientific data *should not* be traded in the same sense as tangible goods, partly in response to the same considerations, but particularly because such trading would run counter to the philosophy of open data [23].

Although scientists undoubtedly do share information without any formal trading arrangement, they are all aware of the disincentives that inhibit the development of a fully open culture. Carter, probing “the economic incentives that motivate the sharing of technical information”, argued that persistence of information provided a strong incentive to exchange [18]. However, Collins had previously attributed a lack of cooperation between universities to their sense of competition [22]. The remarks he quotes are interesting from a behavioural perspective, as is his observation: “Nearly every laboratory expressed a preference for giving information only to those who had something to return”. These conservative attitudes towards true openness are still in evidence today: we have noted how both Downing [13] and Borgman [17] describe attitudes that are inimical to the open sharing of information and data.

A data-sharing model must therefore both provide incentives and overcome the inhibitions introduced by competition. The incentives for any individual scientist might range from increasing the sum of human knowledge to achieving international recognition in one’s field, from self-denying curiosity to naked ambition. Without being cynical, it seems highly unlikely that any scientist would reject recognition for good work done, which leads to the conclusion that appropriate acknowledgement would provide a strong incentive.

A data-sharing model must offer explicit benefits to producers, not only for sharing their data, but also for the effort of curation and creating the metadata. The advantage in sharing data might appear to lie with consumers, but in addition to their obligations to give proper attributions, such as citations, they in turn can become producers who rely on the integrity of other consumers. A data-sharing model based on knowhow trading thus requires an infrastructure that facilitates the formation of social links based on trust, thereby realising the benefits of sharing scientific information and data on much the same basis as technological knowhow.

We believe that a research data exchange with associated services is capable of allaying the main misgivings of practitioners about making data more freely available, and ensure that not only the wider education community but also the public achieves maximum gain from research. To overcome the deterrents identified by Borgman and other observers, the trading infrastructure must ensure transparency, access, acknowledgement, and compliance with conditions. To develop a solution based on a trading environment it is essential that we appreciate fully the obligations that might arise from the practice of trading data in a scientific context, in our case chemistry.

**Fig. 1:**
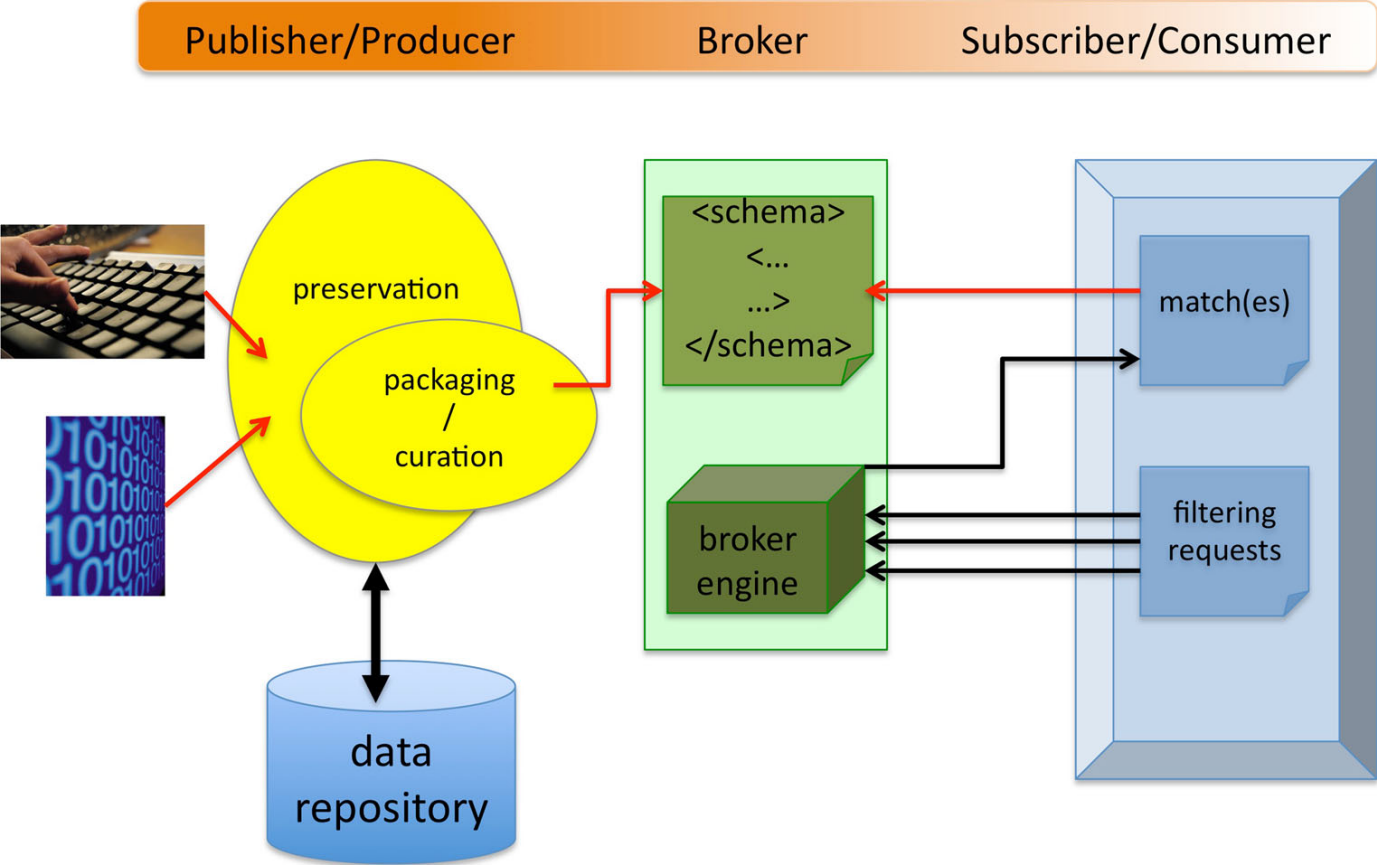
Data sharing exploiting a data broker

For our data- and information-trading environment we envisage deploying a broker to mediate the trading of data, drawing on the parallels between a conventional broker and the Publish/Subscribe methodology to create the required data publication infrastructure, as illustrated in Fig. 1. A key requirement would be to facilitate data discovery by encouraging curation at source. Our perspective takes to a more advanced stage the concept of open repositories by creating an environment, both technical and social, whereby the data in repositories can and will be ‘traded’ and ‘exchanged’. A trading environment therefore supports other initiatives intended to reinforce the collaborative approach and make all the outputs of research discoverable and available for repurposing and reuse in follow-on work: this approach is now almost essential for progress in scientific and other fields of research.

The concept of a notional hub that brings together the disparate facilities comprising the data exchange environment is a valuable aid to understanding the embodiment of a mechanism for sharing data through a trusted broker that we believe has a well-founded analogy with a trading model. Moreover, it is a pivotal point that the hub we envisage is not another data store.

As shown in Fig. 1, the broker would accept packages consisting of metadata conforming to a prescribed schema that describes the nature, provenance, and access provisions for the data being published. The package would *not* include the data itself.

The broker contract would be in the nature of a service level agreement, the provisions of which could include:

- A standard description of each item of data that the broker has available;
- A controlled vocabulary of terms used to classify the data held by the broker;
- Validation of packages on receipt;
- Search facilities for discovering data, not only using the controlled vocabulary but also via free text search;
- A mechanism for querying the provenance of an item or set of items;
- Records of all accesses by consumers;
- Automatic notification to producers of queries and requests, which would form the basis for a reward and recognition system;
- Services, for example, to provide feedback from the consumer to the data producer.

## The challenges

To realise the collaborative environment so vital to the evolution of science and other disciplines, it would be essential to explore existing data sharing practices, and use that insight to understand the deterrents to sharing. The knowledge so gained would inform the development of the tools for realising the open access vision. In addition to the key requirement to understand as fully as possible the incentives and disincentives, we identify the following challenges:

- Enabling open access to data and metadata, including provenance data;
- Enabling publishers to restrict the distribution of the data they share;
- Relieving the burden of curation using, for example, methods as described by Shotton et al. [24];
- Providing access records that researchers can trust and thereby overcome their inhibitions regarding ownership and open access;
- Establishing a trusted reward and recognition system that can recognise use of shared data in the form of a citation, drawing on the experience of organisations such as DataCite [6].

**Fig. 2:**
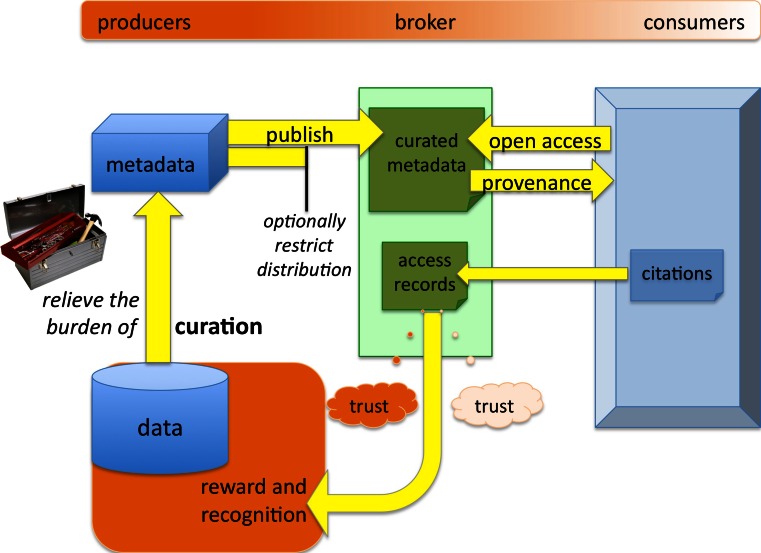
Meeting the challenges of trading and sharing data

Figure 2 illustrates our vision of how these challenges could be met from a trading perspective by adopting an exchange built upon a trusted broker. In summary the brokers and exchanges would help alleviate some of the burden of curation and dissemination from researchers an their institutions and facilitate the maximum traceable impact of their research outputs.

## Conclusions

In the UK the funders of significant government-sponsored research have begun to require explicit data management policies and demand that data be shared as the default strategy. These funders and the Universities and the main recipients of these funds are only just beginning to realize the implications of these demands in terms of the information infrastructure required to collect, retain, curate, and deliver the data (in context and with provenance to meet the requirements of transparency). The researchers’ requirements in terms of reward also need to be considered. If the necessary reward structures are not present then there is no incentive for the researchers to participate beyond what they are contractually obliged to by grant condition; this is not the way to archive the highest quality data and metadata in the public domain.

In this viewpoint article, we suggest that a trading perspective could provide a fruitful area of research, which could benefit from the existence of a proven technology, the trusted broker, on which to base a proof of concept. We suggest that an exchange built upon a trusted broker would make a very valuable component of the national and international data infrastructure and could solve many of the perceived problems in data sharing. We hope by offering this viewpoint to encourage the scientific and technical community to debate the merits of a trading perspective on data sharing and exchange and the potential of trusted brokers and their associated services to promote the open sharing that we believe is so important for future progress.
